# Species delimitation and microalgal cryptic diversity analysis of the genus Micractinium (Chlorophyta)

**DOI:** 10.18699/VJGB-22-11

**Published:** 2022-02

**Authors:** E.S. Krivina, A.D. Temraleeva, Yu.S. Bukin

**Affiliations:** Federal Research Center “Pushchino Scientific Center for Biological Research of the Russian Academy of Sciences”, Pushchino, Moscow region, Russia; Federal Research Center “Pushchino Scientific Center for Biological Research of the Russian Academy of Sciences”, Pushchino, Moscow region, Russia; Limnological Institute of the Siberian Branch of the Russian Academy of Sciences, Irkutsk, Russia

**Keywords:** green microalgae, ABGD, GMYC, PTP, species delimitation, morphology, ecology, phylogeny, 18S−ITS1−5.8S–ITS2 fragment, зеленые микроводоросли, ABGD, GMYC, PTP, морфология, экология, филогения, фрагмент 18S−ITS1−5.8S−ITS2

## Abstract

In this article, the system of the green microalgal genus Micractinium, based on morphological, physiological, ecological and molecular data, is considered. The main diagnostic species characteristics and the taxonomic placement of some taxa are also discussed. Phylogenetic analysis showed that the genus Micractinium is characterized by high cryptic diversity. The algorithms used for species delimitation had different results on the number of potentially species-level clusters allocated. The ABGD method was less “sensitive”. The tree-based approaches GMYC and PTP showed a more feasible taxonomy of the genus Micractinium, being an effective additional tool for distinguishing species. The clustering obtained by the latter two methods is in good congruence with morphological (cell size and shape, ability to form colonies, production of bristles, chloroplast type), physiological (vitamin requirements, reaction to high and low temperatures), molecular (presence of introns, level of genetic differences, presence of CBCs or special features of the secondary structure in ITS1 and ITS2) and ecological characteristics (habitat). The polyphyly of the holotype of the genus M. pusillum as well as M. belenophorum is shown. The intron was effective as an additional tool for distinguishing species, and the results of the intron analysis should be taken into account together with other characteristics. The CBC approach, based on the search for compensatory base changes in conservative ITS2 regions, was successful only for distinguishing cryptic species from “true” members of M. pusillum. Therefore, to distinguish species, it is more effective to take into account all the CBC in ITS1 and ITS2 and analyze characteristic structural differences (molecular signatures) in the secondary structure of internal transcribed spacers. The genetic distances analysis of 18S–ITS1–5.8S–ITS2 nucleotide sequences showed that intraspecific differences in the genus ranged from 0 to 0.5 % and interspecific differences, from 0.6 to 4.7 %. Due to the polyphasic approach, it was possible to characterize 29 clusters and phylogenetic lines at the species level within the genus Micractinium and to make assumptions about the species.

## Abstract (ru)

В статье рассматривается система зеленых микроводорослей рода Micractinium, построенная на основании морфологических, физиологических, экологических и молекулярно-генетических данных. Обсуждаются главные диагностические признаки видов, а также систематическое положение некоторых таксонов. Филогенетический анализ показал, что род Micractinium характеризуется достаточно высоким криптическим разнообразием. Используемые алгоритмы разграничения видов имели различные результаты по количеству выделенных кластеров потенциально видового уровня. Метод ABGD, основанный на дистанциях, является менее «чувствительным». Алгоритмы GMYC и PTP, анализирующие топологию филогенетического дерева, более реалистично отражают систематику рода Micractinium и служат эффективными вспомогательными инструментами для разграничения видов. Кластеризация, полученная двумя последними методами, хорошо согласуется с морфологическими (размеры и форма клеток, способность формировать колонии, продуцирование щетинок, тип хлоропласта), физиологическими (потребность в витаминах, реакция на воздействие высоких и низких температур), молекулярно-генетическими (наличие интронов и их длина, уровень генетических различий, наличие компенсаторных замен (СВС) или характерных особенностей вторичной структуры в ITS1 и ITS2) и экологическими признаками (среда обитания). Показана полифилетичность типового вида рода M. pusillum, а также M. belenophorum. Интрон был эффективен как вспомогательный инструмент для разграничения видов, однако результаты анализа интронов необходимо учитывать в совокупности с другими признаками. Применение СВС-подхода, базирующегося на поиске компенсаторных замен в консервативных регионах ITS2, было успешным только для отграничения криптических видов от «истинных» представителей M. pusillum. При разграничении видов эффективнее учитывать все СВС в ITS1 и ITS2 и анализировать характерные структурные различия (молекулярные подписи) во вторичной структуре внутренних транскрибируемых спейсеров. Анализ генетических дистанций нуклеотидных последовательностей 18S–ITS1–5.8S–ITS2 показал, что внутривидовые различия у представителей рода колебались в пределах 0−0.5 %, межвидовые – 0.6–4.7 %. Благодаря полифазному подходу удалось охарактеризовать 29 кластеров и филогенетических линий видового уровня в рамках рода Micractinium и выдвинуть предположения о видах внутри выделенных групп.Ключевые слова: зеленые микроводоросли; ABGD; GMYC; PTP; морфология; экология; филогения

## Introduction

The genus Micractinium was described by G. Fresenius in 1858 and was referred to the family Micractiniaceae. For a long time, it was thought that this genus includes only microalgae which unlike the genus Chlorella and other ‘small green balls’ form colonies and produce bristles consisting of protein, devoid of cellulose fibers and developing after the formation of a cell wall (Schnepf et al., 1980). The species differences were based on minor changes in the formation of colonies, as well as the length and number of bristles.

Based on the results of the phylogenetic analysis of the 18S rRNA gene, Wolf et al. (2003) concluded that strains of the genus Micractinium are members of the Trebouxiophyceae class and are closely related to the genus Chlorella Beijerinck. Later, Luo et al. (2005, 2006) found that the formation of colonies and the production of bristles is often a reaction to the so-called algophages ‘grazing’ load from (primarily rotifers and ciliates), and in their studies, the authors also suggested that the type species of the genus, M. pusillum, is polyphyletic. Using molecular genetic analysis, Pröschold et al. (2010) proved that the genus Diacanthos with its type species D. belenophorus is a member of the genus Micractinium. Summarizing the results of molecular genetic, morphological, and ontogenetic analyses by Wolf et al. (2003), Krienitz et al. (2004), Fawley et al. (2005), Luo et al. (2010), Pröschold et al. (2010) proposed a new concept of the Chlorella-clade, according to which the genus Micractinium was transferred to the family Chlorellaceae.

Currently, there are 20 species of microalgae in this genus. However, the fragment 18S–ITS1–5.8S–ITS2 was sequenced only for 9 species, among which there are both microalgae with a classical Micractinium-like morphotype, i. e. forming colonies and producing bristles, and organisms with a typical Chlorella-like morphology (for example, M. singularis, M. variabile, M. simplicissimum, M. inermum, M. tetrahymenae, which have single cells and lack bristles under standard conditions) (Hoshina, Fujiwara, 2013; Chae et al., 2019; Pröschold et al., 2020).

Members of the genus Micractinium are widely distributed in various biotopes, including freshwater and brackish water reservoirs, hot springs, and cold waters of Antarctica, at temperatures from zero to above 70 °C (Hoshina, Fujiwara, 2013; Onay et al., 2014; Adar et al., 2016; Chae et al., 2019). They play an important role in the life of ecosystems, actively participating in the processes of photosynthesis of organic substances and photosynthetic aeration, as well as natural self-purification of the reservoir through the accumulation, transformation, and mineralization of pollutants (Vaishlya, Kulyatov, 2011; Mehrabadi et al., 2017). These microalgae are also actively used for the production of animal feed, food additives, and wastewater treatment (Lipstein, Hurwitz, 1983; Onay et al., 2014; Mehrabadi et al., 2017). In addition, some species of the genus Micractinium are recognized as suitable raw materials for biofuels due to a high growth rate combined with a high lipid content (Onay et al., 2014; Adar et al., 2016). Currently, thermophilic and cryotolerant representatives of the genus Micractinium, which are able to accumulate lipids or other valuable substances, are of considerable interest for biotechnology (Onay et al., 2014; Adar et al., 2016; Chae et al., 2021). Accurate species identification, in this case, becomes a priority task, since the ecological plasticity of species to abiotic environmental factors can vary significantly not only within the entire genus but also between closely related species (Onay et al., 2014; Chae et al., 2021).

The goal of this research was a comprehensive study of representatives of the genus Micractinium, including new strains of the Algal Collection of Soil Science Institute (ACSSI), for reliable differentiation of closely related taxa at the species level. For the first time, morphological, physiological, and ecological characteristics were generalized for all the described members of Micractinium, the results of phylogenetic analysis of the 18S–ITS1–5.8S–ITS2 fragment were considered, including the presence of introns and their characteristics, the values of genetic distances, differences in the secondary structures of spacers ITS1 and ITS2, among them the presence of compensatory substitutions (CBC) and structural differences, and species boundaries were determined using GMYC, PTP and ABGD methods. Based on the polyphasic approach, assumptions were made about the criteria for species distinguishing within the genus.

## Materials and methods

Objects of research. The objects of this study were the genetic sequences of strains belonging to the genus Micractinium described and deposited in GenBank, as well as six new strains of microalgae from the ACSSI collection. Strains ACSSI 198, ACSSI 287 and ACSSI 345 were isolated from water from the surface horizon of the pelagic zone of the lake Prudovikov (53°31′44.4″ N, 49°30′58.0″ E, Tolyatti, Samara region, Russia), ACSSI 343 and ACSSI 344 – from water from the surface horizon of the pelagic zone and macrophyte thickets of the lake Bolshoe Vasilyevskoe, respectively (53°32′45.2″ N, 49°32′02.0″ E, Tolyatti, Samara region, Russia). The strain ACSSI 332 (= IPPAS C-16) is a subculture of the IPPAS Collection of microalgae and cyanobacteria, and was isolated from hot springs on the Chukchi Peninsula.

Isolation and cultivation of new strains. A drop of lake water without prefiltration was applied to a solid medium BG-11 with nitrogen (1 % agar, pH = 7.2) and then individual colonies were repeatedly replanted. The obtained isolates were cultured in a climatostat under standard conditions (temperature +23…+25 °C, light 60–75 μM of photons/ (m2·s), photoperiod of 12 hours).

Microscopy. The morphology and life cycle of these strains were studied by light microscopy (light field and interference contrast) using Leica DM750 and Carl Zeiss Axio Scope A1 microscopes (Germany) at the Federal Research Center “Pushchino Scientific Center for Biological Research of the Russian Academy of Sciences”. The results of the observations were documented by working drawings and photographs taken with the help of color digital cameras Videosavr (Russia) and Carl Zeiss MRc 5 (Germany). The follow-up period ranged from 2 weeks to 12 months. To determine the limits of variation of morphological features, the characteristics of 200 vegetative cells of each strain were analyzed

Isolation, amplification, purification, and sequencing of DNA. The total DNA from the strains was isolated using a DNeasy Plant Mini Kit (Qiagen, USA), following the manufacturer’s protocol. For amplification, Screen Mix-HS mixture was used (Eurogen, Russia). Primers for PCR of the 18S and 5.8S rRNA genes and ITS1, ITS2 spacers, and amplification conditions are given in the work of Krivina and Temraleeva (2020). The detection of the target PCR products was carried out electrophoretically in a 1 % agarose gel. For further purification of amplicons from the gel, a Cleanup Standard kit (Eurogen, Russia) was used. The sequencing of the nucleotide sequences was carried out based on CJSC “Syntol” (Russia).

Molecular phylogenetic analysis. To analyze the phylogeny and clarify the taxonomic position of the studied strains, the homology of the nucleotide sequences 18S–ITS1–5.8S– ITS2 was searched using the BLASTn algorithm in GenBank (https://blast.ncbi.nlm.nih.gov). The selection of sequences was carried out based on the criteria of maximum identity (similarity ≥95 %), reading quality, reading length (at least 2300 bp) and belonging to type species and authentic strains.The sample for phylogenetic analysis included 59 strains. The names of taxa are given according to the International Electronic Database AlgaeBase (Guiry M.D., Guiry G.M., 2021). In the BioEdit program, multiple alignment was performed using the ClustalW algorithm. The phylogenetic tree reconstructed by the maximum likelihood (ML) method in the IQ-TREE program (with an assessment of the reliability of the topology by ultra-fast bootstrap analysis and testing of the evolutionary model using the AIC criterion) was used to distinguish species using the Poisson tree processes (PTP) algorithm on an online server https://species.h-its.org/

https://species.h-its.org/

To distinguish species in the data array, the method of automatic search for interspecific gap in genetic distances (automatic barcode gap discovery, ABGD) (Puillandre et al., 2012) was used on an online server https://bioinfo. mnhn.fr/abi/public/abgd/. To analyze ABGD, a matrix of genetic distances calculated using the maximum likelihood method in the IQ-TREE program was used. When using the ABGD method, the results were analyzed both in the initial partition mode and in the recursive partition mode. The third method was a generalized mixed Yule model taking into account the integrity of species (general mixed Yule coalescent model, GMYC) (Fujisawa, Barraclough, 2013), implemented in the ‘splits’ package for the R programming language v. 3.4.4 (https://www.R-project.org/). For GMYC analysis, an ultrametric tree reconstructed in the BEAST v. 1.10.4 program was used.

https://bioinfo. mnhn.fr/abi/public/abgd/

https://www.R-project.org/

The reconstruction of the tree in BEAST was carried out using four speciation models: the Yule speciation model (Aldous, 2001) with a strict molecular clock; the Yule speciation model with a relaxed molecular clock with evolution rates distributed according to a lognormal distribution; the birth–death speciation model (Lambert, Stadler, 2013) with a strict molecular clock; a model of speciation of the birth–death of species with a relaxed molecular clock with the rates of evolution distributed according to the lognormal distribution. The selection of the best speciation model was carried out by comparing the marginal likelihood values calculated by the method of sequential sampling (Lartillot, Philippe, 2006) in the BEAST v. 1.10.4 program. During the reconstruction of the tree, the BEAST program set 50,000,000 generations for Markov chains and 250,000 generations of Markov chains and 200 steps for calculating the marginal likelihood. With these parameters of the number of generations, all the values of the ESS statistics (the convergence indicator of the BEAST analysis) were more than 200. An ultrametric Bayesian phylogenetic tree was used to visualize the analysis results. A sample of ultra-fast bootstrap analysis trees obtained in the IQ-TREE program was combined with the topology of a Bayesian ultrametric tree to calculate bootstrap supports by the maximum likelihood method (ultra-fast bootstrap analysis). Thus, the support of the ultrametric Bayesian tree topology was evaluated by the Bayesian inference (BI) using a posterior probabilities and bootstrap analysis. To calculate bootstrap supports, we used an algorithm previously developed by us (Temraleeva et al., 2018), implemented using the functions of the APE package (Paradis et al., 2004) for the R statistical software environment v. 3.4.4. A representative of the sister genus Chlorella (Trebouxiophyceae, Chlorophyta), C. vulgaris, was chosen as an outgroup during phylogenetic reconstructions. The distribution of genetic distances was visualized as a histogram in the R statistical software environment v. 3.4.4.

Genetic differences between nucleotide sequences were characterized using genetic distances (K2P distances), which were calculated in the MEGA 6.0 program. The boxplot of genetic distances was built in the R statistical software environment v. 3.4.4 (https://www.R-project.org/). To compare the topology of trees, we used data from articles (Krienitz et al., 2004; Luo et al., 2006; Hoshina et al., 2010, 2017; Pröschold et al., 2010, 2011, 2020; Bock et al., 2011; Hoshina, Nakada, 2018).

Folding of ITS1 and ITS2 was performed using the RNAfold web server (http://rna.tbi.univie.ac.at//cgi-bin/ RNAWebSuite/RNAfold.cgi) in accordance with the principle of minimum energy. When assessing the correctness of the prediction of the secondary structure, ITS1 and ITS2 were guided by A. Coleman (2015) and Caisová et al. (2013), respectively. The comparison of the secondary structure of spacers between strains, the search for conservative motives and compensatory substitutions (CBCs) was carried out in the 4SALE program (Seibel et al., 2008). In the analysis of ITS2 for the species distinguishing, special attention is paid to the approach of sensu A. Coleman (2000, 2015), according to which the presence of even one CBC in conservative regions of ITS2 (5 bp of I helix, 10 bp of II helix, all III helix) in two microalgae correlates with their sexual incompatibility. The secondary structures of spacers are visualized in the PseudoViewer3 program.

Statistical analysis of various characteristics of representatives of the genus Micractinium. For comparative analysis, the characteristics of the strains were encoded in the form of binary vectors. The length of the binary vector of the analyzed feature was equal to the number of its possible states, while each element corresponded to a certain state. For the analyzed strains, 1 was recorded in the position corresponding to the state of the characteristic, the remaining elements had the value 0. All binary vectors determining the states of each of the traits for all the strains studied in the analysis were summarized in a single table. The analysis used strains for which the states of 80 % or more of the considered traits were known, the remaining strains were excluded from the analysis.

On the basis of a binary table of feature states, the similarity and difference of strains were visualized using multidimensional scaling, for which a matrix of Jacquard distances was used (one minus the share of common nonzero states in the total number of non-zero states in the two strains being compared), during the calculation of which for each pair of strains, features that were indeterminate for one of the strains were excluded. In order to determine the significance of a trait in the overall distribution of distances between strains, the Mantel test (Mantel, 1967) was used based on the Pearson correlation coefficient, the reliability of the correlation was determined by a permutation test (10,000 permutations). During the Mantel test, the general matrix of Jacquard distances was compared with the distance matrices calculated for each feature separately. The higher the value of the Pearson correlation coefficient for the trait under consideration, the greater the contribution it makes to the separation of strains. All calculations were performed using the functions of the ‘vegan’ package (Dixon, 2003) for the R statistical software environment.

## Results

Morphology of ACSSI strains. All studied strains had a Chlorella-like morphotype: the cells were single, spherical in shape, without bristles. The vegetative cell sizes of ACSSI 343, ACSSI 344, and ACSSI 345 were more than ACSSI 198, ACSSI 287, and ACSSI 332 (Table 1).

**Table 1.**
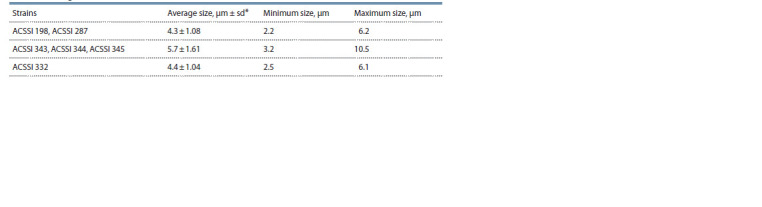
The vegetative cell sizes of the studied strains * sd – standard deviation. 200 measurements for each strain.

The chloroplast is parietal, mainly cup-shaped. However, in the strains ACSSI 343, ACSSI 344 and ACSSI 345, a saucer-shaped chloroplast (~20 %) and a hollow spherical with a hole (~20 %) are found in adult cells. The pyrenoid is single, spherical or broadly oval with a starch sheath. All strains reproduce by autospores. The number of autospores in the strains ACSSI 343, ACSSI 344, and ACSSI 345 varies from 2 to 8, in the strains ACSSI 198, ACSSI 287, as a rule, 2–4 autospores were noted (8 autospores are rare), while in ACSSI 332, there were no more than 4. Based on the morphological characteristics, the studied strains were initially assigned to the genus Chlorella.

**Fig. 1.**
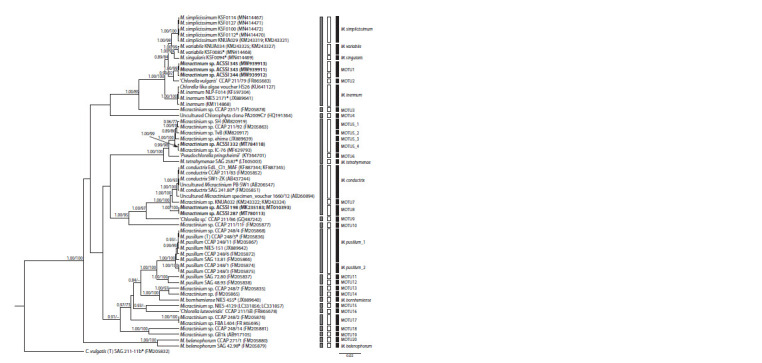
A rooted ultrametric phylogenetic tree of green microalgae of the genus Micractinium, constructed by the Bayes inference (BI), based on the 18S–ITS1–5.8S–ITS2 sequences (2546 bp). As statistical support for the nodes of the tree, a posterior probabilities (PP) and bootstrap values (BP), respectively, are indicated; the values of PP < 0.7 and BP < 70 % are not shown. The model of nucleotide substitutions: GTR + I + G. ACSSI strains are highlighted in bold; * – authentic strains; (T) – type species. The rectangles indicate clustering by various methods of distinguishing species: gray – ABGD, white – PTP, black – GMYC.

Phylogenetic analysis. The best model of DNA evolution for the studied dataset of nucleotide sequences (18S–ITS1– 5.8S–ITS2) is GTR + I + G (AIC = 38198.0101), which was used for all further calculations. The results of the “path sampling” analysis showed that the best model for the reconstruction of the phylogenetic tree by the Bayesian method in the BEAST program is a model of speciation of the birth–death of species with a relaxed molecular clock with the rates of evolution distributed according to the lognormal distribution (the lowest value of the marginal likelihood index Ln(L) = –18996.049). The phylogenetic tree reconstructed according to this speciation model was used for further analysis. Fundamental differences between the topology of the BEAST tree (Fig. 1) and the topology of the IQ-TREE (Suppl. Material 1)1 were not detected in nodes with high support.

Supplementary Materials are available in the online version of the paper: http://vavilov.elpub.ru/jour/manager/files/Suppl_Krivina_Engl.pdf


According to the results of the analysis, all six strains belonged to the genus Micractinium (see Fig. 1). Strains ACSSI 343, ACSSI 344, and ACSSI 345 with high statistical supports (posterior probabilities PP = 0.89, bootstrap probabilities BP = 84 %) are combined with single-celled, nonbristle- producing species M. simplicissimum, M. variabile, M. singularis, and the strain CCAP 211/79. The sister to them is M. inermum (PP = 1.00, BP = 100 %). The level of genetic differences between the strains ACSSI 343, ACSSI 344, ACSSI 345, and sister clusters was 0.7–0.9 %. The strain ACSSI 332 with maximum statistical support was clustered with Chlorella-like strains TvB, SH, CCAP 211/92, ehime, IC-80. The sister phylogenetic lines to this cluster are the incorrectly identified Pseudochlorella pringsheimii and M. tetrahymenae (PP = 0.99–1.00, BP = 98–100 %). The strain ACSSI 332 does not have an intron of the 18S rRNA gene, unlike TvB, SH, CCAP 211/92. The genetic distances between it and the strains TvB, SH, CCAP 211/92, ehime, IC-80 varied in the range of 0.1–0.5 %, with P. pringsheimii and M. tetrahymenae – 1.1–1.2 %. The sister strains to ACSSI 198, 287 are M. conductrix and the KNUA032 strain with a Chlorella-like morphotype (statistical support is maximum). The level of genetic differences ranged from 0.7 to 1.3 %.

The secondary structure of ITS1 and ITS2. The length of ITS1 of the studied strains was 238–267 nt, ITS2 – 242– 243 nt. The ITS1 and ITS2 secondary structures generally corresponded to the models proposed by Coleman (2000, 2015) for eukaryotic organisms. The strains ACSSI 343, ACSSI 344, and ACSSI 345 had 1 CBC in the III helix of ITS1 compared to M. inermum, 1 CBC in the conservative region of II helix and 1 CBC in the variable IV helix of ITS2 compared to M. simplicissimum. Strains ACSSI 332, 198, 287 did not have CBC compared to similar species. However, the strains ACSSI 198, 287 differed in structure of ITS2 helix II from M. conductrix. The mismatch in its upper part of strains ACSSI 198, 287 consisted of 4 unpaired nucleotides, and of M. conductrix – of 10.

**Fig. 2.**
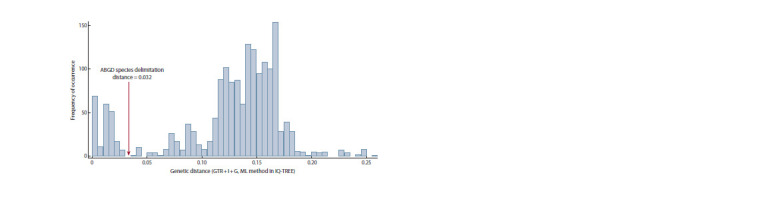
Histogram of the distribution of genetic distances between representatives of the genus Micractinium The species delimitation boundary determined by the ABGD method is shown.

Delimitation of species. The ABGD method of species delimitation identified 18 MOTUs (molecular operational taxonomic units) of the species level in the genus Micractinium, not counting the external group. The ABGD distance of species differentiation in the pairwise comparison of sequences was 0.032 (Fig. 2). The results of ABGD analysis in the range of species distinction distances according to the variants of the algorithm of initial delimitation (initial partition) and recursive delimitation (recursive partition) coincided with each other.

Using the GMYC method, the largest number of clusters of the species level was identified – 33 (the delimitation distance of species is 0.0015). Statistical support for the results of differentiation P = 1.07493e–07 < 0.05, therefore, there is enough data in the array to obtain reliable results. Using the PTP method, 30 species were identified, which is close to the results of the GMYC method. The results of species differentiation by ABGD, GMYC, and PTP methods are shown on the phylogenetic tree (see Fig. 1). All clusters of the species level identified by these methods have high statistical support (PP = 0.95–1.00, BP = 90–100 %).

**Fig. 3.**
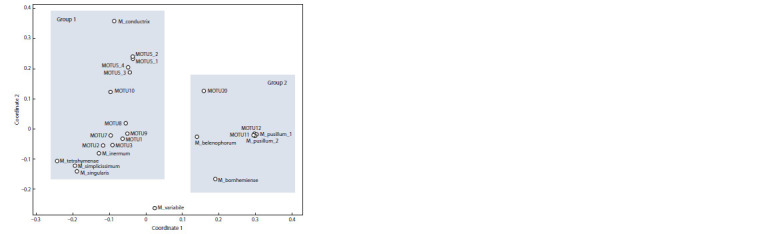
The dot – MOTUs scattering diagram in the space of two coordinates of multidimensional scaling, constructed on the basis of similarity and difference of strains by a set of features.

Multidimensional scaling. To clarify the taxonomic status, we correlated the MOTUs isolated by the GMYC algorithm with their morphological, physiological, ecological, and molecular genetic characteristics (Suppl. Material 2). It should be noted that during the multidimensional scaling only the presence of an intron was taken into account from the genetic characteristics, while the remaining parameters are discussed separately. According to the results of the analysis, the studied MOTUs were divided into two groups (Fig. 3).

Group 1 included strains with single cells that do not produce bristles. Within it, only representatives of MOTU5_1, MOTU5_2 were united into one subgroup. Members of the other species/MOTU had a unique position. It should be noted separately that the studied strains ACSSI 343, ACSSI 344, ACSSI 345 (MOTU1), and strains ACSSI 198, ACSSI 287 (MOTU8) did not form a single complex with related species according to the results of phylogenetic analysis. The ACSSI strain 332 and IC-80 (MOTU5_4) are localized next to the ehime strain (MOTU5_3), while representatives of MOTU5_1 and MOTU5_2 are somewhat removed

All representatives of Group 2, on the contrary, have bristles and, as a rule, form colonies. The strains initially identified as M. pusillum formed a single group. All the other species were quite distant from each other. An intermediate position between the groups is occupied by M. variabile, in which only a part of the population is able to produce bristles and form colonies in the presence of algophages. According to the results of the Mantel test, in addition to the ability to produce bristles and form colonies, chloroplast type, intron number, reproduction type, cell maximum size and shape, and lifestyle were considered significant features when distinguishing MOTUs (Table 2).

**Table2.**
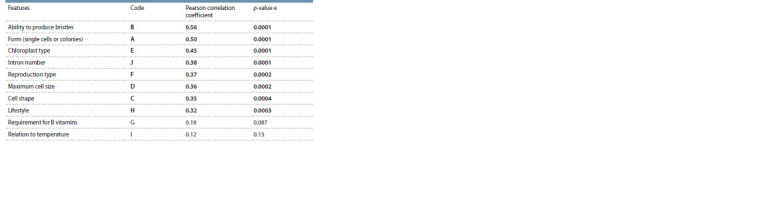
Mantel test results Notе. Statistically signif icant features are highlighted in bold.

## Discussion

The ABGD method, based on the analysis of genetic distance matrices, compared with other methods, identified the smallest number of MOTUs of the species level with the greatest distance of their differentiation. This is consistent with the research of Zou et al. (2016a, b), who also noted a lower sensitivity of this method. The GMYC and PTP algorithms, using phylogenetic trees as initial data, are able to capture the features of genetic divergence between strains, identify a larger number of putative species, and are more consistent with the modern concept of the genus Micractinium. To clarify the taxonomic status of MOTU, the results of the GMYC algorithm, which identified the largest number of potential species, were correlated with their morphological, physiological, ecological, and molecular genetic characteristics.

Morphological characteristics. All representatives of the genus Micractinium, for which morphological characteristics are known, had a number of common features: a coccoid thallome, one parietal chloroplast, one pyrenoid with fragmented starch sheath, asexual reproduction by autospores. Important morphological criteria are the ability to produce bristles and form colonies (Krienitz et al., 2004; Luo et al., 2006). According to these characteristics, 2 morphotypes can be distinguished within this genus: Chlorella-like (under standard conditions, single cells do not produce bristles) and Micractinium-like (single cells or colonies producing bristles), which is confirmed by the results of multidimensional scaling (see Fig. 3). Other significant morphological characteristics for representatives of the genus Micractinium are chloroplast type, maximum size and shape of cells. However, Micractinium morphology is rather poor. The study did not reveal a single feature that could be considered as a universal tool for species distinguishing. For example, the phenotypes of M. simplicissimum and M. singularis are extremely similar, and it is quite problematic to separate them morphologically. It should be noted that microalgae of genus Micractinium have high phenotypic plasticity, and their morphotype can vary depending on the “grazing” load from algophages (Krienitz et al., 2004; Luo et al., 2006). Thus, in some cells of M. variabile, which usually exhibits a Chlorella-like morphotype, the formation of colonies and the production of bristles is noted at high trophic pressure of algophages (Chae et al., 2019). At the same time, representatives of M. pusillum, M. bornhemiense, M. belenophorum can stop producing bristles and form colonies during prolonged cultivation, especially on solid agar (Krienitz et al., 2004; Luo et al., 2006).

Reproduction. The main reproduction type of members of the genus Micractinium is asexual with the help of autospores. At the moment, an exception is M. pusillum strains that reproduce using oogamy (sexual process) (Krienitz et al., 2004; Luo et al., 2006). However, according to the whole genome analysis, meiotic genes, the presence of which suggests a sexual process, were found in many representatives of the Trebouxiophyceae class, for which only asexual reproduction was observed (Fučíková et al., 2015). This question is still open and needs to be studied for members of Micractinium.

The vitamins requirement and lifestyle. Most of the species are free-living organisms and vitamins do not need to be added when culturing them under laboratory conditions. At the same time, a specific feature of M. conductrix is the requirement for vitamins B1 and B12 for normal implementation of vital processes. This species is an obligate endosymbiont and naturally receives vitamins from the host organism (Vorobyev et al., 2009; Hoshina et al., 2010; Pröschold et al., 2011). It is noteworthy that other obligate endosymbionts of the clade Chlorella (С. variabilis, Carolibrandtia ciliaticola) also grow only on media enriched with vitamins (Pröschold et al., 2011; Hoshina et al., 2017; Hoshina, Nakada, 2018). The strain CCAP 211/11F isolated from lichen and the facultative endosymbiont M. tetrahymenae are also cultivated on media containing B1 and B12. However, there is no information that vital activity of the strains is not possible without them. According to the results of multidimensional scaling, lifestyle is one of the significant characteristics when distinguishing species, while the need for B vitamins is a highly specific property characteristic only of M. conductrix. However, it is a unique feature of this species and helps to separate the representatives of this species from the “sister” ones at the cultivation stage.

Temperature. In relation to temperature, members of the genus Micractinium, for the most part, show mesophilic characteristics (Hong et al., 2015). However, M. simplicissimum, M. variabile, M. singularis and the strain of Micractinium sp. KNUA032 withstand the effects of low temperatures. They are able to survive and reproduce at temperatures up to +5 °C, showing their cryotolerant properties. One of the main adaptation strategies of these microalgae species is to maintain vital activity through the accumulation of unsaturated fatty acids (Hong et al., 2015; Chae et al., 2019). The strains TvB, SH, CCAP 211/92 are thermophiles that can withstand high temperatures (Adar et al., 2016). The ACSSI 332 strain is presumably resistant to high temperatures, since it was isolated from a hot source. The question of the thermophilicity of related strains ehime and IC-76 remains unexplored. Multidimensional scaling has shown that resistance to the effects of extremely low or high temperatures are specific properties characteristic of only a small number of species. However, such species have a great biotechnological potential, and therefore they need to be carefully studied (Onay et al., 2014; Adar et al., 2016; Chae et al., 2021).

Intron. As an auxiliary tool for distinguishing species, it was effective in proving the species status of strain KNUA032, ACSSI 198 and ACSSI 287 cluster, and representatives of M. conductrix, all strains of which have an intron 324 nt long in the 18S rRNA gene. The composition of this intron and its specific position in 18S rRNA have been repeatedly considered by researchers as a characteristic feature of this species (Vorobyev et al., 2009; Hoshina et al., 2010; Spanner et al., 2020). The intron was also useful in distinguishing between the strains of M. belenophorum: CCAP strain 271/1, in contrast to the authentic strain SAG 42.98, has an intron 315 nt long in the 18S rRNA gene. At the same time, the presence of introns in some species may indicate that speciation processes began but are still occurring at the population level (Goankar et al., 2018). For example, within the clade Chlorella Hoshina et al. (2021) found in some populations of C. variabilis, geographically isolated from each other, the length of introns can vary. Within the genus Micractinium, a similar situation can be observed among MOTU5 members with similar morphology, genetic distances at the intraspecific level and without CBC (see Suppl. Material 2). In the strains TvB, SH (MOTU5_1), CCAP 211/92 (MOTU5_2), unlike the related ehime (MOTU5_3), ACSSI 332, and IC-76 (MOTU5_4), an intron with a length of 351 nt is present in the 18S rRNA gene. In other words, although the intron is a statistically significant feature in the differentiation of MOTUs, it cannot be used as the main criterion for the division of species, but only as an auxiliary one.

Comparative analysis of the secondary structure of internal transcribed spacers. The application of the sensu Coleman (2000, 2015) CBC approach, based on the search for CBC exclusively in conservative ITS2 regions, was successful in distinguishing the strains SAG 48.93 and SAG 72.80 from the “true” representatives of M. pusillum, M. conductrix from the KNUA032 strain, as well as the authentic strain M. belenophorum SAG 42.98 compared to strain CCAP 271/1. The low efficiency of the sensu Coleman CBC approach for distinguishing green microalgae species with low genetic divergence was also noted in (Hoshina, Fujiwara, 2013; Song et al., 2018). Therefore, at present, when distinguishing species of the genus Micractinium, all CBCs in ITS1 and ITS2 are often taken into account (Hoshina, Fujiwara, 2013; Chae et al., 2019; Pröschold et al., 2020). However, for example, between the species M. singularis and M. variabile, there are no CBCs in both ITS1 and ITS2. At the same time, Chae et al. (2019) noted that these species differ in the structure of the ITS2 helix I.

The use of characteristic structural differences in the secondary structure of internal transcribed spacers as an analogue of CBC among members of the genus Micractinium was first proposed by Hoshina et al. (2010), who found a specific feature in the M. conductrix ITS2 secondary structure. In all representatives of the clade Chlorella in general and the genus Micractinium in particular, the II helix of ITS2 consists of two double-stranded regions articulated by an “elbow-like bulge”. Compared to other species, M. conductrix has a large “elbow” of 10 unpaired nucleotides (bachelor nucleotides), although other species have from three to six unpaired nucleotides. We believe that this feature can be considered a “molecular signature” of M. conductrix. For comparison, the sister strains KNUA032, ACSSI 198, and ACSSI 287 had only four unpaired nucleotides in this region. Thus, the CBC approach is not a universal tool for distinguishing species of the genus Micractinium. In addition, when analyzing internal transcribed spacers, one should not limit oneself only to searching for CBC in conservative areas, it is also important to take into account the structural features of their secondary structures

**Fig. 4.**
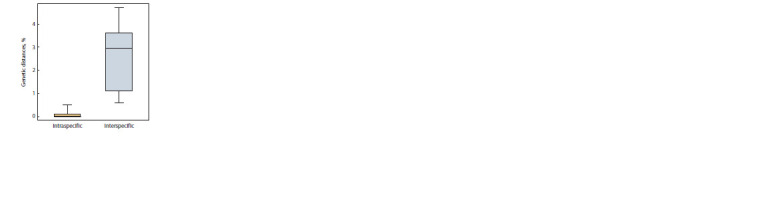
Genetic distances within the genus Micractinium The borders of the box show the first and third quartiles, the bold horizontal line – the median value, the “whiskers” – the span.

Genetic distances. A comparative analysis of the level of genetic differences of the fragment 18S–ITS1–5.8S–ITS2 of the studied strains with such diacritical features as the cell shape and size, the ability to produce bristles, the chloroplast type, the intron presence in the 18S rRNA gene, CBC in ITS1 and ITS2, molecular signatures, the ratio to temperature, vitamin requirement, lifestyle, clustering by ABGD, GMYC, PTP, allowed us to clarify intraspecific and interspecific levels genetic differences (Fig. 4). Within the species, the genetic distances varied in the range of 0–0.5 %, between species – 0.6–4.7 %. Minimal genetic distances were observed between single-celled and non-bristle-producing cryotolerant Antarctic species M. singularis and M. variabile, which, under the influence of “grazing” load, is able to form colonies and release bristles. The maximum genetic distances are between the Chlorella-like cryotolerant M. simplicissimum and M. bornhemiense, which under standard conditions has a classical Micractinium-like morphotype.

**Fig. 5.**
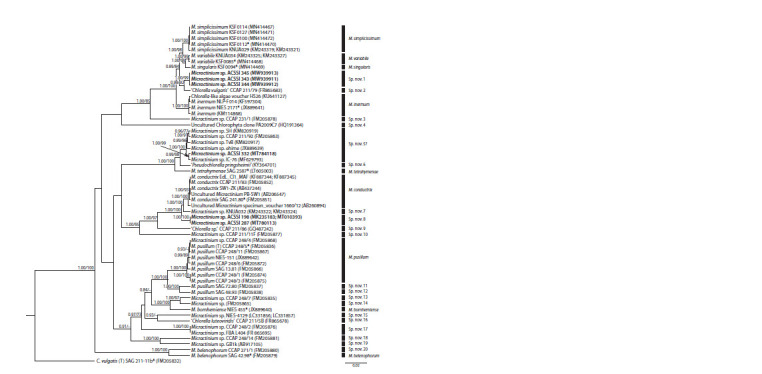
The proposed separation of species within the genus Micractinium based on a comprehensive analysis of features. The ACSSI strains studied in this work are highlighted in bold; * – authentic strains; (T) – type species; ? – taxonomic status needs to be clarif ied.

Based on the results of a comprehensive analysis of the above parameters, 29 species were identified within the genus Micractinium (Fig. 5), including candidates for three new species from the ACSSI Algological Collection, whose validation is yet to be performed.

## Conclusion

At present, only 9 species were described in the genus Micractinium using a combination of morphological and molecular genetic methods, but according to the analysis results, its true species richness turned out to be significantly higher – at least 29 species. The delimitation method ABGD, which is based on a matrix of genetic distances, is less “sensitive” and identified only 18 MOTUs of the species level, while the more advanced topological algorithms GMYC and PTP found 33 and 30, respectively. In our opinion, GMYC and PTP reflect the taxonomy of the genus Micractinium more realistically, being an effective auxiliary tool for distinguishing species

Multidimensional scaling of qualitative characteristics of the strains under consideration showed that the most significant for representatives of the genus Micractinium is the ability to produce bristles and form colonies, the chloroplast type, the intron presence, the reproduction type, the cell maximum size and shape, and lifestyle. However, not a single trait has been identified that could be considered as a universal species criterion. The requirements for B vitamins and resistance to extremely low or high temperatures are highly specific properties that are characteristic of only a small number of species and help in distinguishing them from “sister” species. The application of the CBC approach based on the search for CBC in conservative ITS2 regions was successful only for the separation of “true” representatives of cryptic species (SAG 48.93, SAG 72.80) from M. pusillum, M. conductrix from strain KNUA032 and M. belenophorum from strain CCAP 271/1. When analyzing ITS1 and ITS2, in addition to searching for CBC, the structural features of their secondary structures should be taken into account. Based on the results of the analysis of the genetic distances of the 18S–ITS1–5.8S–ITS2 nucleotide sequences, it can be assumed that intraspecific differences are in the range of 0–0.5 %, interspecific differences are in the range of 0.6–4.7 %.

## Conflict of interest

The authors declare no conflict of interest.
